# Mycobiota of Potato-Cereal Soft Wraps and the Production Facility

**DOI:** 10.3390/foods12173238

**Published:** 2023-08-28

**Authors:** Cathrine Kure Finne

**Affiliations:** Nofima-Norwegian Institute of Food, Fisheries and Aquaculture Research, P.O. Box 210, N-1431 Ås, Norway; cathrine.finne@nofima.no

**Keywords:** mould, spoilage, *Penicillium commune*, mycobiota, wraps

## Abstract

The aim of this study was to investigate the mycobiota of potato-cereal soft wraps and in the processing area. Potato-cereal soft wraps are cooked, cold-stored and mashed potatoes that are kneaded together, usually with wheat flour, to form dough. In order to identify the main spoilage mould of this product, 150 visible mould colonies from mouldy wraps were identified. Five different mould species were isolated; *Aspergillus niger*, *Penicillium brevicompactum*, *Penicillium commune*, *Penicllium corylophilum* and *Pencillium discolor*. The dominating spoilage mould was *Penicillium commune* with 83.9% of the colonies. In order to study the mycobiota of the production area, 271 samples of air and surfaces were collected. In total, 647 mould colonies were isolated from air and surface samples. The mycobiota of air consisted of 27 different species within 9 different genera, and the mycobiota of surfaces consisted of 14 species within 4 different genera. *Penicllium* species were the dominating genera both in air and on surfaces, and *Penicillium commune* was the dominating species in the processing environment as well. *Penicillium commune* was found in the bakery and also in other production rooms. Spores from the flour and from soil on potatoes can disperse in the air as aerosols and may contaminate the wraps after baking when the product is cooled before packaging.

## 1. Introduction

Lompe and Lefse are potato-cereal soft wraps readily available in most Norwegian supermarkets and are an increasingly popular consumer choice. They are a commercial product in Norway and are a type of unleavened potato-cereal flour, tortilla-like soft wrap originated in Scandinavia during the 19th century [1]. Lompe is also consumed in some of the Northern states of the USA. It is often used in a variety of sweet or savoury dishes, the most common of which in Norway is as a wrap for various cooked sausages/hot dogs.

In the normal production process of Lompe and Lefse (hereafter named potato-cereal soft wraps or only wraps), firstly cooked, cold-stored and mashed potatoes are kneaded together, usually with wheat flour, to form dough. Moisture present in the potatoes is usually sufficient to form dough, but water and/or potato flakes can be added if necessary [2]. From similar dough, potato-based unleavened bakery products are made, such as Italian potato-based pasta (gnocchi) [3]. 

Producers of these potato-cereal soft wraps in Norway receive returned products due to visible mould spots, and this has significant economic consequences. 

In certain periods, there are many packages with visible mould spots; however, there are only sporadically mouldy products returned to the producer for the most part. If mould spots are observed on the products, the wraps are not eaten and become waste. Reduction in the mould spoilage of the wraps will therefore produce less food waste and prolong shelf life. In order to prevent and reduce food waste and reduce the cost, it is important to identify the contamination source and identify preventative measures. 

Filamentous fungi can synthesize and accumulate primary and secondary metabolites [4], and together with bacteria and plants, they are among the most prolific producers of secondary metabolites [5]. Secondary metabolites are derived from four chemical classes: polyketides, non-ribosomal peptides, terpenes and indole alkaloids. Due to this, the mould species produce off-flavours and -odours, discolouration and rotting; some produce secondary metabolites that are mycotoxins. 

Normally less than 10, and often only one to three species, are responsible for mould spoilage of a food product, and the critical species are often completely different for each food type [6]. This is due to a complicated combination of biotic and abiotic factors. Identification of the contamination source and prevention of mould spoilage of food products therefore requires knowledge of the associated mycobiota of the specific food product to identify the source of the contamination in the processing of each product. 

*Penicillium* and *Aspergillus* species are the main spoilage genera of bread and other bakery products [7,8,9]. Different species within these genera such as *P. roqueforti*, *P. paneum*, *P. polonicum*, *P. galdbrum*, *A. flavus* and *A. niger* spoil the products. The main contamination source of bakery products are spores from raw materials that are dispersed in facilities as aerosols. Even though most of the spores are inactivated in the baking step, the spores in the air may contaminate the final products [7,10,11]. To my knowledge, there are no scientific reports available on the identity of moulds from potato-cereal soft wraps.

In order to reduce the spoilage and waste of potato-cereal soft wraps with mould spots and identify preventive measures, the aim of this study, therefore, was to identify the main spoilage moulds on the potato-cereal soft wrap with visible mould spots, identify the main sources of these moulds in the production facility and suggest preventative measures.

## 2. Materials and Methods

### 2.1. Potato-Cereal Soft Wrap Production

The potato-cereal soft wrap is commercially made by rolling potato-cereal flour dough into thin sheets (ca. 2 mm), cutting it into a flat oval or round shape, before baking it by dry convection heat on a griddle (ca. 15 s each side at 225 °C). After the dough is baked, it is transported on a belt to cool down and is then exposed to air. Usually, several wraps are placed in a pile and packaged in plastic for distribution. 

### 2.2. Isolation of Moulds from Product

Potato-cereal soft wraps with visible mould spots were collected from a Norwegian producer during a period of approximately 15 months, from October 2019 to January 2021. The products in unopened plastic bags were returned from the stores to the producer due to visible mould on the product. A total of 50 packages with mouldy wraps were received in the laboratory. Each package contained 10 potato-cereal soft wraps. The pH in the wraps was 5.3–5.5 (827 pH lab, Metrohm, Switzerland). 

A small opening in the plastic package was made with a sterile scalpel above the visible mould spot. A small section of the mould colonies was plated in 9-cm diameter Petri dishes with malt extract agar (MEA) (Oxoid, Hampshire, United Kingdom) and incubated for 7 days at 25 °C. A total of 150 visible mould colonies were picked and isolated. The Petri dishes were inspected, and the colonies were subcultured as described in Section 2.4.

### 2.3. Isolation of Moulds from Air and Surface Samples 

Air in the production facility and surfaces of equipment in the bakery were sampled 15 and 7 times, respectively, from October 2019 to April 2020. Table 1 shows the sampling points. The production facility included rooms for potato storage, a potato washing room, a room for the peeling and mashing of potatoes and the bakery. Sampling was performed in October and December in 2019 and January, February and April in 2020.

Air sampling was performed by exposing Petri dishes (9 cm in diameter) containing Dichloran 18% glycerol agar (DG18) (Oxoid, Hampshire, United Kingdom)with supplements as described in Samson et al. 2010 [12,13] for 1 h. The Petri dishes were placed horizontally and without cover. The agar plates were incubated in darkness for 7 days at 25 °C and the colonies were inspected and counted. Representatives for each of the different colonies were subcultured onto MEA.

Surface sampling of equipment was performed by swabbing approximately 100 cm^2^ with a sterile cotton swab before the swab was streaked onto MEA plates. The agar plates were incubated in darkness for 7 days at 25 °C and the colonies were inspected. Representatives for each of the different colonies were subcultured onto MEA.

### 2.4. Subculturing and Identification of Moulds

The mould colonies were subcultured onto MEA agar plates with supplements [13]. *Penicillium* species were also plated onto czapek yeast extract agar (CYA) [13], yeast extract sucrose agar (YES) [13] and creatine sucrose agar (CREA) [14], based on morphological characteristics on agar plates, and three-point inoculated and incubated for 7 days. MEA and YES at 25 °C, CYA at 25 °C and 30 °C and CREA at 20 °C. 

The moulds were identified based on a morphology according to Samson et al. 2010, Samson et al. 2004 and Crous et al. 2007 [15,16,17]. For identification of *Penicillium commune* and *Penicillium palitans*, a filter paper method was used according to Lund [18].

A representative number of each of the different mould types were identified to species levels using ITS sequencing with primers ITS1/ITS4 [19], and β-tubulin primers Bt2a/Bt2b [20] in addition for identification of *Penicllium* [15,21].

## 3. Results

### 3.1. Mycobiota of Potato-Cereal Soft Wrap

An overview of the mould species isolated from potato-cereal soft wraps with visible mould colonies are shown in Table 2. Six different mould species were isolated and identified, five of these were *Pencillium* species and one *Aspergillus* species. The dominating mould species was *P. commune* with 83.9% of the colonies. The other species isolated were other *Penicillium* species and *Aspergillus niger*. *Penicillium commune* dominated the products during the whole period.

### 3.2. Mycobiota in Air and Surfaces in the Bakery

A total number of 271 samples were collected from air in the production facility and from surfaces in the bakery. The sampling points are described in Table 1. Moulds were detected on 105 (51%) of the air samples and 34 (53%) of the surface samples. Approximately half of the air samples (48%) from the bakery contained moulds, compared to 73% of the air samples from other rooms. 

The number of mould colonies on the air samples were generally lower in the room with production line 3 than in the room with production line 1 and 2 (Table 3), although the number of mould colonies from the air samples were constant during the period of sampling. 

The mycobiota of air were identified. In total, ten different genera were isolated, and *Penicillium* were the dominating genera with 69% of the colonies (Table 3). A total of 31 different species were isolated from air. The bakery with production line 1 and 2 were not physically separated, but there still were higher levels of *Cladosporium* and *Aspergillus* in the air around production line 2 compared to line 1 and higher frequency of *Pencillium* in the air around production line 1. The most frequently isolated species from all air samples was *Penicillium commune*. *Pencillium brevicompactum* and *P. nalgiovence* were also dominant in the air in other rooms. 

Four different genera were isolated from surfaces. *Pencillium* was the most frequently isolated genera. The most frequently isolated species was *P. commune* in the air and on surfaces in the bakery (Table 3 and Table 4). The level of *P. commune* varied with higher levels in the air at some of the sampling days (Table 5) and corresponded to a generally higher level of moulds in air at the same days. *Penicillium commune* was generally higher in the air in the room with production line 1 and 2 compared to the level in the room with production line 3.

## 4. Discussion

Visible mould spots were isolated from Norwegian potato-cereal wraps produced during a period of 15 months. The most frequently isolated mould species from wraps was *P. commune* with 83.9% of the colonies. Hence, this is the major spoilage mould of this product, which is important knowledge for the identification of contamination sources and recommendation of preventative measures. *Penicllium commune* is commonly found in arctic and temperate regions and is commonly found on cereal-based products [9] as well as on cheese [22,23], dried meat, rye bread and in soil [15]. *Penicllium commune* is known to produce many different metabolites such as cyclopenin, cyclopiazonic acid, palitanin, rugulovasin A and B, viridicatin, viridicatol and chromanols [15,24]; hence, it has the potential to produce changed flavour and taste in the wraps. Some of these secondary metabolites are toxic. From the producers’ experience, the consumers do not eat the wraps when they are mouldy, and the mould spoilage of the wraps, therefore, do not represent a food safety issue. 

Other mould species such as *P. brevicompactum*, *P. roqueforti*, *P. discolor*, *P. corylophilum* and *Aspergillus niger* were also isolated from the mouldy wraps; however, only in a low percentage of the products. These species are commonly found in an indoor environment, and *P. brevicompactum* and *P. roqueforti* are some of the main spoilage moulds on bread [8,9]. 

In order to study the mycobiota of air and surfaces and identify the contamination source of the spoilage mould, samples were collected on 15 different production days. Out of 271 samples, moulds were detected on 51%. This indicates that moulds are distributed in the facility, both in the bakery and in other rooms. The mycobiota of air and surfaces consisted of a large number of different mould species, 27 different species within 9 different genera in air, and 14 species within 4 different genera on surfaces. 

The major spoilage mould of wraps, *Penicillium commune,* was the most frequently isolated species from the air as well, comprising 32% of the isolated colonies (Table 3). Hence, the spoilage mould on the product was also dominant in the air in the production facility. The majority of *P. commune* in the air was isolated from samples in the bakery on sampling days with a high number of moulds. The level of moulds was higher at some of the sampling days. For two of these sampling days, the increased level of moulds in the facility was due to a high level of moulds in the air in the potato washing room. At two of the other sampling days, with a high level of moulds in the facility, it was due to a generally higher level of mould spores at most of the sampling point. 

It seems like there is an increase in *P. commune* at specific days, both in the air and on surfaces. The reason for this is not clear from the results; however, 36% of the *P. commune* isolates were from other rooms like the potato washing and cooking rooms, even though the number of samples from the air of these rooms were fewer than the number of samples from the bakery. The reason for the increased level of *P. commune* at specific sampling days could be due to the increased level of *P. commune* spore transmission with raw materials, wheat flour and potatoes. Mould spores can be introduced in the production facility from flour and potatoes with soil on its surfaces and be dispersed in the air as aerosols [7,25]. This may therefore contaminate the wraps after baking when the product is cooled before packaging. Spores in the potato mash are believed to be inactivated in the baking process [26]. The contamination of the wraps occurs, therefore, after baking and before packaging when the wraps are cooled. Mould from mouldy wraps that are returned to the producers could be a source of the increased level of *P. commune* in the facility; however, they are in plastic packages, and its less likely that spores from mouldy products would be spread in the facility. Nevertheless, packages with mouldy wraps need to be handled very carefully in order to prevent spoilage mould from spreading to the air in the production facility.

Different species of *Cladosporium* and *Aspergillus* were also frequently isolated from the air. Both genera are frequently isolated from indoor air, production facilities and from food [15]; however, they do not form the spoilage mould of the wraps in the presented study. Compared to the air in the processing of cheese and meat, there is less diversity of moulds species in the bakery [27,28].

Thirteen species within four genera were isolated from different surfaces in the bakery. *Penicillium commune*, the main spoilage mould of wraps, was isolated from conveyor belts before packaging and from grabs that pick up potato-cereal wraps and place ten in a stack (before transporting them to be packaged in plastic). There is no step after the baking that removes mould spores from the surface of the product; consequently, the *P. commune* spores grow on the product later when exposed to air. *Pencillium commune* is not regularly found on surfaces in contact with the wraps, but when they are, they may contaminate the product and produce mould spots later if exposed to air. Consequently, it is important that surfaces such as transport belts, grabs, etc., are cleaned properly. 

There are no barriers in the wrap production facility that prevent spores from being spread between different rooms. Establishment of zones and higher air pressure in the bakery is important in order to prevent mould spores from being distributed to the bakery. The results in the present study show high levels of moulds in the potato storage and washing rooms. Therefore, the producer should consider either washing the potatoes in a separate room before they are transported into the production building for storage and cooking or receiving washed potatoes from the farmers. This will probably reduce the general level of mould spores in the facility, and also lower the level of spoilage mould in the air. 

The level of *P. commune* spores in the air needs to be as low as possible in order to prevent spores on surfaces that are in direct contact with the products. One can believe that when the producer sporadically received returned products with mould spots, they were contaminated with *P. commune* from the air. This is due to the fact that spores from the air only contaminate some of the wraps. In other periods, the producers received many returned products with mould spots in a short period of time, which indicates that there are surfaces with *P. commune* that contaminate a large number of wraps, such as the surfaces of grabs that were contaminated with *P. commune* spores in this study.

Even if hygienic zones are established, appropriate cleaning is conducted and potato washing is completed before they are taken in to the production facility, it may be difficult to reduce the level of mould spores to a satisfying level. Organic acid and their salts are often used as preservatives in bakery products to avoid fungal deterioration of the products. However, the efficiency of the organic acids is greatly influenced by the pH of the products [29,30,31] and efficiency is highest at a low pH. The pH in the wraps in this study (5.3–5.5) is close to the pH where acid efficiency decreases; hence, mould may still grow and result in spots on the wraps. Different disinfection technologies can be used for the cleaning of surfaces [32]. Fogging disinfection with the use of a hydrogen peroxide (H_2_O_2_) mist has shown to be effective in the reduction of mould spores on surfaces [33].

To inactivate spores that have settled on the surface of the wraps during cooling after baking, UV-treatment of the surface can be included in the production line before the packaging. UV-lamps are used on bread-slicing machines and in tunnels for baked bread [34], and this extends the shelf life of the product. 

Identification of the spoilage mould on a species level, based on morphological characterisation and ITS-sequencing, is not sufficient to answer the question of the spreading of mould spores in the facility. Spoilage mould needs to be identified below species levels to answer if there are persistent strains that contaminate the products or if there are new strains regularly introduced in the facility [21,35] that contaminate the product and cause visible mould spots. Different molecular typing methods have been used [36]; using whole genome sequencing to distinguish among the *P. commune* strains might be a useful method [37]. Application of metabolomic chemotaxomic in fungal taxonomic identification has progressed considerably [38]. In future studies, *P. commune* isolates from products and from the processing area in this study should be further identified on the strain level using molecular techniques as whole genome sequencing or metabolomic chemotaxonomic in order to understand the spreading of the spoilage strains of *P. commune* in the facility.

## Figures and Tables

**Table 1:** Description of the sampling point of air and surfaces, the number of samples and number of samples with moulds.

Sampling Point	No. of Samples	No. of Samples with Moulds
Air		
Potato washing and cooking room	10	9
Cooling room	6	3
Peeling room	2	2
Vacuum cleaner room	2	1
Storage room for plastic	2	2
Elevator from first to second floor	2	1
Eating room for workers	2	1
Bakery production line 1	54	28
Bakery production line 2	52	28
Bakery production line 3	75	30
Total number of samples of air	207	105
Surfaces in bakery		
Production line 1		
Conveyor belt before frying	3	3
Conveyor belt after frying	9	8
Conveyor belt before packaging	2	1
Production line 1 and 2		
Conveyor belt in robot	2	1
Grabs in robot	13	6
Conveyor belt before packaging	4	2
Conveyor belt after packaging	3	1
Production line 2		
Conveyor belt before frying	3	1
Conveyor belt after frying	5	4
Production line 3		
Conveyor belt before frying	2	1
Conveyor belt after frying	8	3
Conveyor belt before packaging	6	1
Conveyor belt after packaging	4	2
Total number of samples of surfaces	64	34
Total number of samples	271	139

**Table 2:** Mould species isolated from potato-cereal soft wraps as a percentage of the total number of colonies identified.

Mould Species	Percentage
*Aspergillus niger*	0.9
*Penicillium brevicompactum*	7.1
*Penicillium commune*	83.9
*Pencillium corylophilum*	5.4
*Penicillium discolor*	0.9
*Penicillium roqueforti*	1.8

150 colonies in total.

**Table 3:** Mould species isolated from samples of air and number of colonies of each species from the bakery and other rooms.

	No. of Colonies from Air Samples				
	Bakery				
Mould Species	Production Line 1	Production Line 2	Production Line 3	Other Room	Total
*Acremonium* sp.	1			2	3
*Aspergillus cancicus*	1				1
*A. flavus*	3	10			13
*A. fumigatus*		5			5
*A. niger*	5	24	28	5	62
*A. versicolor*		4			4
*Aureobasium pullulans*		3			3
*Cladosporium cladosporioides*	3	45	1	3	52
*Cladosporium* sp.	6	7	1	17	31
*Mucor plumbeus*	3	1		2	6
*Paecilomyces variotii*				1	1
*Rhizopus oligosporus*				1	1
*Phoma glomerata*				17	17
*Penicillium alii*	8				8
*P. aurantiogriseum*				2	2
*P. brevicompactum*	5		1	33	39
*P. chrysogenum*	4	5	20	3	32
*P. cyclopium*	5	6			11
*P. crustosum*		2	3	3	8
*P. commune*	57	42	34	74	207
*P. decumbens*			1		1
*P. expansum*	1	1			2
*P. freii*		5			5
*P. nalgiovense*	5			26	31
*P. polonicum*		1			1
*P. roqueforti*		13			13
*Penicillium* sp.	56	19	7	6	88
Total no colonies	163	193	96	195	647

**Table 4:** Mycobiota of surface samples in the bakery.

		Sampling Point Detected		
Mould Species	Production Line 1	Production Line 1 and 2	Production Line 2	Production Line 3
*Aspergillus* sp.		Grabs in robot		
*Aspergillus candidus*	Conveyorbelt before frying			
*A. niger*		Grabs in robot	Conveyorbelt after frying	
*Cladosporium* sp.				Conveyorbelt
*C. cladosporiodies*	Conveyorbelt before frying			
*Penicillium brevicompactum*	Conveyorbelt after frying	Conveyorbelt before packaging		Packaging machine
*P. camemberti*			Conveyerbelt after frying	
*P. chrysogenum*		Conveyerbelt in robot	Conveyerbelt after frying	
*P. commune*	Conveyorbelt before packaging Conveyorbelt	Conveyorbelt before packaging Packaging machine Conveyerbelt in robot Grabs in robot	Conveyorbelt	
*P. corylophilum*		Conveyorbelt after robot		
*P. crustosum*		Conveyerbelt in robot		
*P. decumbens*		Conveyorbelt after robot	Conveyerbelt	Packaging machine
*Penicillium* sp.	Conveyorbelt Conveyorbelt before robot	Grabs in robot		Conveyerbelt before packaging
*Trichoderma* sp.				Packaging machine

**Table 5:** Sampling points with *P. commune* detected. Numbers of *P. commune* colonies on air-and samples.

Sampling Point Air	No. of Colonies of *P. commune* Detected at Different Sampling Day ^1^															Total No. of Colonies
	1	2	3	4	5	6	7	8	9	10	11	12	13	14	15	
Potatowashing and cooking room		12														12
Vaccumcleaner room		1														1
Elevator from 1. to second floor	15															15
Production room line 1		1		1	4	2		36	20	3	5	6	10		25	113
Production room line 2				4	3	1	2	10	3	25	8		20		30	106
Production room line 3			3								2	25	6		2	38
Total number of samples	15	14	3	5	7	3	2	46	23	28	15	31	36		57	285

^1^ Not detected = blank cells.

## Data Availability

The data used to support the findings of this study can be made available by the corresponding author upon request.
